# Individual differences in brain attention networks: the challenge of indexing temporal change

**DOI:** 10.3389/fcogn.2025.1547773

**Published:** 2025-06-18

**Authors:** Gerald Matthews, Almira Kustubayeva, Manzura Zholdassova, Gulnur Borbassova

**Affiliations:** ^1^Department of Psychology, George Mason University, Fairfax, VA, United States; ^2^Department of Biophysics, Biomedicine, and Neuroscience, Center for Cognitive Neuroscience, Al-Farabi Kazakh National University, Almaty, Kazakhstan

**Keywords:** attention networks, sustained attention, alerting, event-related potentials, individual differences, affect, cognitive neuroscience

## Abstract

The vigilance decrement in speed and accuracy of response is prevalent in studies of sustained attention. The amplitudes of Event-Related Potentials (ERPs) elicited by task stimuli also show temporal decline. However, it is difficult to link the behavioral performance decrement to loss of efficiency in the specific brain circuits that control human attention. A recent study published by the authors used an extended duration-version of the Attention Network Test to explore temporal changes in behavioral and electroencephalographic indices in executive control, alerting, and orienting attention networks. This study found evidence for temporal decline in ERPs associated with the alerting network, as well as slowing of uncued reaction time. This study, like most psychophysiological studies of sustained attention, analyzed group data. The present article provides new analyses of data from the authors' previous study to investigate individual differences in loss of attention on the extended ANT, and their relationships with positive and negative affect. Data analyses addressed the temporal stability of attention network metrics, inter-relationships between different metrics, and associations between metrics and affective states. Results illustrated some challenges in assessment of brain networks at the individual level on tasks requiring sustained attention. Issues included differential temporal stability of metrics, divergence of behavioral and ERP measures, and distinguishing changes in network function from changes in baseline response. The ANT is well-supported by group data as a tool for investigating attentional functioning. However, the present results suggest that caution is necessary in utilizing network indices at the individual level in clinical and other applied contexts.

## 1 Introduction

The Attention Network Test (ANT; Fan et al., 2002) is widely used in diverse research areas including studies of cognitive neuroscience, human performance, and clinical conditions (de Souza Almeida et al., 2021; Posner, 2023). A strength of the ANT is that is based on Petersen and Posner's (2012) attention network theory which distinguishes brain circuits for executive control, alerting, and spatial orienting. The theory is supported by converging evidence from behavioral, psychophysiological, and neurological research (Petersen and Posner, 2012; Posner, 2023; Posner and Rothbart, 2007).

Much research using the ANT is focused on group data, such as differences between patient groups and healthy controls. However, the ANT is considered a validated psychometric test (Fan et al., 2002, 2007) although some psychometric limitations have been identified (MacLeod et al., 2010). As such, it can be utilized to assess attentional functioning in individuals, supported by correlational evidence that establishes a nomological network for the three indices of network functioning (e.g., Matthews and Zeidner, 2012). In this article, we consider the extent to which behavioral and electroencephalographic ANT indices can serve as indicators for individual readiness for vigilance and sustained attention.

### 1.1 Attentional network theory and research

According to attention network theory, the executive control network, localized in prefrontal and frontal areas, supports top-down regulation of attention, especially when stimuli elicit conflicting responses (Posner and Rothbart, 2007). Alerting is supported by frontal, parietal, and thalamic circuits that maintain attention following arrival of a warning of cue stimulus. Orienting, supported by parietal areas and the frontal eye fields, enhances selective attention to a sensory source. The ANT (Fan et al., 2002) measures the three networks as follows. The basic task is to discriminate whether an arrow stimulus is pointing left or right. Executive control is measured as the difference in response times (RTs) to target stimuli flanked by incongruent or congruent stimuli. Inhibition of incongruent flankers engages the executive control network. Alerting is measured as the RT difference for cued and uncued stimuli, with a variable foreperiod. Assessment of orienting is based on response to stimuli presented above or below the initial fixation point. The orienting index is the RT difference between trials with a spatial cue to direct attention and trials with a non-directional cue presented at fixation. The three indices are largely independent of one another, although dependencies that reflect interactions between the three networks may occur (Fan et al., 2002, 2007). Indeed, brain networks defined by intrinsic connectivity may overlap and it is challenging to map attention networks; the Petersen and Posner (2012) theory may need updating (Markett et al., 2022).

The ANT has been used to investigate attentional dysfunction in a wide range of clinical conditions, including psychiatric disorders such as attention deficit/hyperactivity disorder (ADHD; Arora et al., 2020), depression (Sinha et al., 2022), anxiety (Heeren et al., 2015), neurodegenerative diseases (Sarrias-Arrabal et al., 2023), and physical conditions such as cardiovascular disease (Razumnikova et al., 2021). Use of the ANT can link disorders to specific networks, such as deficits in executive control observed in schizophrenia (Spagna et al., 2018), depression (Sinha et al., 2022), and anxiety (Pacheco-Unguetti et al., 2011). The ANT may thus be useful to clinicians for psychiatric and neurological assessment, identifying cognitive decline, and as a diagnostic aid (Guo et al., 2022; Li et al., 2023).

The ANT has also been used in studies of individual differences in performance in non-clinical samples. Such studies elucidate relationships between personality traits, mood states and attentional processing (Matthews and Zeidner, 2012; Moriya and Tanno, 2009; Noh et al., 2012). For example, Matthews and Zeidner (2012) found that extraversion and conscientiousness were associated with stronger executive control. Some ANT studies investigated individual differences in attentional functioning in applied contexts. These include attentional factors in vehicle driving performance (Guinosso et al., 2016; López-Ramón et al., 2011) and impacts of shift work (Sumińska et al., 2021).

Studies of the ANT and individual differences in affect illustrate the challenges of finding replicable effects. Theories of affect propose that executive control is associated with negative emotional conditions including anxiety (Eysenck et al., 2023) and depression (Quigley et al., 2022). Matthews and Zeidner (2012) confirmed that state distress was related to impaired executive control, but other studies have failed to find similar associations (Finucane et al., 2010; Noh et al., 2012). Another study found that trait but not state anxiety was associated with poorer executive control (Pacheco-Unguetti et al., 2010; Study 1).

Negative affect (especially anxiety) is linked to vigilance for threat, so that associations with alerting and orienting are expected (Ghassemzadeh et al., 2019). Pacheco-Unguetti et al. (2010) confirmed this hypothesis for orienting but not alerting. Several studies have reported associations between stronger orienting and various trait and state negative affect dimensions (Matthews and Zeidner, 2012; Moriya, 2018; Noh et al., 2012; Pacheco-Unguetti et al., 2010), although there are also contrary findings (Moriya and Tanno, 2009). There are similar inconsistencies in studies of alerting, with some studies showing a positive association between the ANT index and negative affect scales, and some showing no relationship (Moriya, 2018). Noh et al. (2012) reported a negative association between alerting and positive affect.

The ANT can also be used to secure electrophysiological as well as behavioral indices of attentional network activity. Analysis of the Event-Related Potential (ERP) response to ANT stimuli may provide a more sensitive indicator of network activity than RT-based indices, as well as tracking the time course of activity (Kustubayeva et al., 2022; Neuhaus et al., 2010). Typically, the cueing manipulations associated with alerting and orienting networks increase the amplitude of the early N100 wave at posterior sites, whereas the incongruent flankers that elicit executive control reduce the later P300 response (Galvao-Carmona et al., 2014; Kaufman et al., 2016; Kustubayeva et al., 2022; Neuhaus et al., 2010). There has been extensive research on the use of ERPs for evaluating neurocognitive functioning in a range of applied contexts (Fu and Parasuraman, 2006), usages that might be enhanced by further research on the validity of ERPs as measures of brain attention networks.

Use of the ANT for diagnostic purposes in applied contexts requires that is suitable for assessing the individual. Some psychometric concerns have been raised including relatively low reliability of measures (Ishigami and Klein, 2010; MacLeod et al., 2010), statistical interdependence of network metrics (MacLeod et al., 2010), handling of errors (de Souza Almeida et al., 2021), and choice of control condition trials (de Souza Almeida et al., 2021). An analysis of fMRI data secured from an ANT study raised similar concerns about reliability and discrimination of attentional networks (Kong et al., 2024). The use of difference scores as network indices is potentially a source of difficulty, especially if criterion values are associated with RTs in control or baseline conditions. Similarly, when using the ANT to investigate temporal performance decrements, change scores may be confounded with baseline scores. Typically, psychophysiological data obey the Law of Initial Value (LIV; Wilder, 1957), such that change is negatively correlated with baseline value, potentially leading to artifactual findings (Burt and Obradović, 2013). There are different methods for calculating change scores. The most straightforward is to calculate a simple difference or delta between the index measured at baseline and measured during task performance, or, as in the present study, between the index measured at early and late temporal stages. Alternative metrics for change include percentage change from the initial score difference and residualized change score. Different metrics have differing psychometric properties and differing correlations with other criterion variables (Burt and Obradović, 2013; Matthews et al., 2017a).

### 1.2 Applications to sustained attention and vigilance

The ANT is a promising tool for understanding sustained attention and the vigilance decrement, i.e., the decline in target detections characteristic of performance over extended time intervals (Warm et al., 2008). Currently, the leading theory of vigilance is based on attentional resource theory (Helton and Wen, 2023; Neigel et al., 2020; Warm et al., 2008), although the decrement has also been attributed to failures in executive control (Luna et al., 2021; Thomson et al., 2015) and to attentional lapses (Smallwood et al., 2004). However, a limitation of resource theory is that “resources” are difficult to define and operationalize precisely (Matthews et al., 2000). The Petersen and Posner (2012) theory potentially supports a more precise account of which specific attention network(s) become impaired during prolonged performance. A case could be made for each of the networks contributing to loss of target detection accuracy. Thomson et al. (2015) stated that impairment in executive control leads to failure to allocate resources effectively, without temporal change in resource availability. Posner (2008) himself attributed vigilance to the alerting network. Consistent with this view, vigilance is impaired by introducing temporal asynchrony to the stimulus sequence (Scerbo et al., 1986), so that there is a variable rather than a constant foreperiod, increasing demands on alerting. Conversely, providing a cue that signals likely arrival of a target reduces vigilance decrement (Hitchcock et al., 2003). The role of orienting has been neglected but an earlier generation of theories of vigilance including expectancy and observing theory highlighted the possible role of selective attention failures in decrement (Davies and Parasuraman, 1982). Spatial uncertainty in multiple modalities also amplifies vigilance decrement (Hess and Greenlee, 2024).

ANT studies of sustained performance have provided mixed outcomes. Zholdassova et al. (2021, Study 1) investigated the sensitivity of the three attentional networks to temporal change by utilizing an ANT that contained three times as many trials as the standard ANT, lasting about 1 h. There were improvements in alerting and executive control in initial trial blocks, after which performance tended to stabilize; no network exhibited temporal decrement. Zholdassova et al. (2021, Study 2) introduced masking and trial-blocking manipulations to increase task demands, as suggested by a resource theory perspective. However, even under increased task demands no temporal decrements in the ANT indices were found. This study found that RTs for congruent-flanker trials tended to increase over time, highlighting the issue that changes in ANT indices may potentially be contaminated by changes in baseline RTs. Overall, these studies suggested that all three networks were resilient during prolonged performance and the traditional vigilance decrement (Davies and Parasuraman, 1982; Warm et al., 2008) could not be attributed to any specific network.

An alternate approach is to modify the ANT to measure vigilance independently of the three Petersen and Posner (2012) networks. The ANTI-VEA (Luna et al., 2022) interpolates additional trials to measure “executive vigilance” to small vertical displacements of the central target arrow, and “arousal vigilance” to a decrementing time counter. Executive vigilance represents the ability to monitor and detect infrequent critical signals, similar to classic vigilance tasks such as the Mackworth Clock (Luna et al., 2023). Arousal vigilance reflects readiness for rapid response without the need for the response selection, similar to the Psychomotor Vigilance Task (PVT; Drummond et al., 2005). Two reports on a large-scale study (Luna et al., 2021, 2022) reported temporal decrements on both these novel indices of vigilance.

Luna et al. (2022) also found an increase in the standard ANT executive control index across the first two three trial blocks of their 33 min task, contrary to Zholdassova et al.'s (2021) finding. Participants who showed decreasing executive control on the standard ANT index also showed greater temporal decrement on the novel executive vigilance metric. These findings were interpreted as supporting resource-control theory (Thomson et al., 2015), which attributes vigilance decrement to weakening executive control over time. Luna et al. (2022) also showed increasing RTs on arousal vigilance trials, although the arousal vigilance decrement was not modulated by executive control. Studies using the ANTI-VEA have also investigated factors that influence vigilance such as caffeine and exercise (Sanchis et al., 2020). Further discussion of ANTI-VEA research is beyond the present scope but it appears that there may be multiple mechanisms controlling temporal decrements on the ANT, depending on the task version used.

Given the challenges of demonstrating temporal decrements on the standard ANT indices, Kustubayeva et al. (2022) investigated whether ERPs might provide more sensitive measures of temporal change in the Petersen and Posner (2012) networks. Previous studies of vigilance and other tasks requiring sustained attention typically show temporal decline in the amplitude of both N100 and P300 waves (Arnau et al., 2021) although there have been some contrary findings (e.g., Haubert et al., 2018). An ANTI-VEA study (Luna et al., 2023) found that temporal decrement in executive vigilance was accompanied by increased amplitude in parietal P300 response on correct detection trials but there were no significant temporal changes in either behavioral or ERP responses for arousal vigilance. Kustubayeva et al. (2022) recorded ERPs from multiple sites during performance of the extended-duration version of the ANT. Behavioral data showed a tendency for RT to increase in most conditions. There was no significant temporal change in executive control over time but, paradoxically, the alerting index showed increasing values over time. This finding reflected a greater slowing in the control, no cue condition (ΔRT = 28 ms) compared with the double-cue condition that activates alerting (ΔRT = 14 ms). Participants become more dependent on the cue over time as in standard vigilance paradigms (Hitchcock et al., 2003) but the Alerting index was misleading as an index of temporal change.

Kustubayeva et al. (2022) also found a general tendency for N100 and P300 amplitudes to decline over time in multiple conditions, consistent with previous ERP studies of sustained attention (Arnau et al., 2021). Temporal declines in amplitude were somewhat similar for congruent and incongruent flanker trials suggesting that there was no decrement specific to the executive control network. However, N100 amplitude decrement at posterior sites in the double cue condition was about twice the magnitude of the decrement in the no-cue condition. These data suggest temporal deterioration in alerting over time, but the ERP data did not exactly match the behavioral data.

### 1.3 The present study

Kustubayeva et al. (2022) confirmed that, in ANT data, ERP data may provide a more sensitive test of temporal change in network function than the Fan et al. (2002) behavioral indices. The present study reports additional analyses of the Kustubayeva et al. dataset to examine the psychometric properties of indices of temporal change in behavioral and ERP metrics for the three attentional networks. What do the various metrics available tell us about the individual's neurocognitive readiness for performing sustained attention tasks?

Specifically, data were analyzed to address the following issues:

- *Stability of individual differences*. We assessed the test-retest stability of behavioral and electroencephalographic ANT metrics across the first and last stages of the 70-min task. Metrics included Fan et al.'s (2002) indices for the three networks, as well as a baseline response index, given evidence for temporal change in RT and ERP amplitude in control conditions (Kustubayeva et al., 2022; Zholdassova et al., 2021). Given that ANT metrics correlate with various stable personality traits (e.g., Matthews and Zeidner, 2012) and clinical conditions (e.g., Sinha et al., 2022), we hypothesized that both types of metric would show significant stability. From an applied perspective, results indicate whether initial levels of performance and ERP amplitude predict responses toward the end of the task.
- *Inter-relationship of behavioral and ERP metrics*. We expected that RT and ERP-based metrics for the same network would intercorrelate. We also checked correlations between N100 and P300 amplitudes. These reflect different cognitive processes and so should dissociate.
- *Inter-relationships of initial and change scores*. There are two conflicting perspectives on the relationship between initial metric values and subsequent temporal change. Consider an individual whose alerting network is already functioning poorly at the beginning of the task. Given the general trend toward temporal decline in alerting, the person may show a larger decline in alerting metrics than someone with a well-functioning network. That is, initial processing inefficiency predicts greater vulnerability to subsequent impairment. (The logic is similar to additive factors theory, which posits that two sources of impairment will together impact performance disproportionately: Verwey et al., 2015). Conversely, adopting the LIV perspective suggests that an individual initially poor in alerting will show relatively small subsequent decline, either for statistical reasons including regression to the mean or because there is less scope for decline relative to a high-alerting individual.
- *Correlations between change metrics*. Analyses of change scores compared metrics based on a simple difference score or delta, with residualized metrics that express the extent to which the change score is more or less than expected on the basis of initial level of the metric. Although these two metrics typically correlate highly they may have different psychometric properties and relationships with external criteria (Burt and Obradović, 2013; Matthews et al., 2017b). We examined the inter-correlations of behavioral and ERP-based change metrics for both types of change score, on an exploratory basis.
- *Correlations with individual differences in affect*. Previous work has shown correlations between the ANT RT-based indices and affective variables. Findings are mixed but scales for negative affect tend to correlate with impaired executive control and enhanced orienting (Matthews and Zeidner, 2012; Moriya, 2018; Noh et al., 2012; Pacheco-Unguetti et al., 2010). We explored whether scales for positive and negative affect might relate more strongly to ERP than to behavioral measures. We also tested associations with baseline RT, which may relate more strongly to affective state than the network indices do (Finucane et al., 2010). We also explored relationships between affect scales and network change metrics, given that sustained attention impairments may provide a marker for various clinical conditions including mood disorder (Fortenbaugh et al., 2018).

## 2 Method

### 2.1 Participants

One hundred and two healthy volunteers (49 males; 53 females) were recruited from the Almaty city area and universities. Mean age was 22.33 (SD = 5.63). Achieved power values for Pearson correlations of 0.1, 0.3, and 0.5 (*p* < 0.05, two-tailed) were 0.17, 0.87, and 1.00. The study received approval from the Ethics Committee of the Faculty of Medicine and Health Care of the al-Farabi Kazakh National University.

### 2.2 ANT task

The ANT task was programmed in E-Prime 2.0 software (Psychology Software Tools, Pittsburgh, PA). The task was identical to Fan et al.'s (2002) ANT, except that the number of trials was extended to nine periods of 96 trials (864 in total). Task duration was ~70 min. The display for the task was 65 cm from the participant's eyes. Stimuli were presented in black, against a white background (see Kustubayeva et al., 2022, for illustrations). Cue stimuli subtended ~0.3° visual angle, and the arrow stimuli ~0.5°. After presenting an initial fixation cross and a cue stimulus, a central arrow stimulus (the target) appeared on the screen. The participant had to discriminate the direction of the arrow as quickly and as accurately as possible by using left and right response keys. The experiment included four categories of the cue—no cue, double cue, central cue, spatial cue (above or below fixation cross); and three categories of the flanker—congruent, incongruent, or neutral. The task was programmed as a sequence of nine blocks of trials. Each block contained two repetitions of 48 different trial types (96 trials per block), i.e., 4 cue types × 3 flanker types × 2 target locations (above or below fixation) × 2 target directions (left or right). All stimulus attributes were counter-balanced in a fixed pseudorandom sequence. Response time to target in ms and accuracy were recorded for each trial.

Kustubayeva et al. (2022) analyzed data using means averaged across three consecutive stages, i.e., blocks 1–3, 4–6, and 7–9. This article compared data from the first and third stages to investigate temporal changes. Attentional network indices were calculated according to Fan et al.'s (2002) formulae:

Executive control = mean RT for incongruent flanking trials – mean RT for congruent flanking trials;

Alerting = mean RT for no-cue trials – mean RT for double-cue trials;

Orienting = mean RT of center cue trials – mean RT of spatial cue trials.

Mean RT on trials with no cue and neutral flankers was used to provide a baseline measure of response speed. As a check on the consistency of using the mean to capture central tendency in response, we calculated the alpha coefficient for the six RTs that are utilized in the Fan et al. (2002) formulae. Alphas were 0.989 at stage 1, and 0.986 at stage 3.

### 2.3 EEG recording

EEG recording was done with a 256 Hz sampling rate with a Neuron-Spectrum_4 system (Neurosoft Ltd, Ivanovo, Russia). Electrodes were placed according to the 10–20% international recording system from frontal, temporal, parietal, occipital, and central areas (FPz, F3, F4, F7, F8, Fz, FCz, C3, C4, Cz, CPz, P3, P4, Pz, O1, O2, Oz) with indifferent ear electrodes in the following situations: open eyes (1 min); closed eyes (1 min); completing the ANT task (70 min). EEG/ERP preprocessing and analysis of P300 and N100 parameters for the central and parietal-occipital electrodes (Fz, Cz, Pz, FPz, FCz, CPz, P3, P4, O1, O2) were done with the EEG/ERPlab toolbox (Lopez-Calderon and Lusk, 2014). Preprocessing included DC correction, bandpass filtering (0.1–30 Hz), epoching, baseline correction, artifact rejection (75uV), and artifact removal with ICA algorithm (Independent Component Analysis). N100 wave was defined as the maximum negative peak at the latency from 240 to 290 ms after target stimulus; P300 wave was determined as the maximum positive peak between the latencies 250 and 600 ms. Latencies were determined separately for each participant by calculating the peak amplitude for each participant in each condition. These intervals were chosen to capture peak amplitudes across multiple recording sites at intervals consistent with previous ERP studies of the ANT (e.g., Neuhaus et al., 2010). Further details of EEG recording methods are available in Kustubayeva et al. (2022).

ERP data were analyzed by defining sets of electrodes linked to each of the three networks according to past practice in ERP studies and theory (Petersen and Posner, 2012). For the executive control network, we averaged ERP amplitudes at Pz and CPz sites, following Kaufman et al. (2016). For alerting and orienting networks, amplitudes were averaged across Pz, P3, P4, O1, and O2 electrode sites (Neuhaus et al., 2010).

Network indices for ERP N100 and P300 amplitudes were calculated according to Kaufman et al.'s (2016) formulae, analogous to the behavioral ANT indices:

Executive control = ERP amplitude for congruent flanking trials – ERP amplitude for incongruent flanking trials;

Alerting = ERP amplitude for double-cue trials – ERP amplitude for no-cue trials;

Orienting = ERP amplitude of spatial cue trials – ERP amplitude of center cue trials.

Because N100 is a negative voltage, we multiplied N100 amplitude measures by −1 so that higher scores indicate a larger-amplitude wave.

We also analyzed no-cue baseline measures of amplitude equivalent to the behavioral baseline measure. As for RTs, we calculated alpha coefficients for the six amplitude measures utilized in the above formulae. For N100, alpha was 0.953 at stage 1 and 0.933 at stage 3. For P300, the respective alphas were 0.961 and 0.953.

### 2.4 Measure of affect

The Positive and Negative Affect Scale (PANAS; Watson et al., 1988) was used to measure positive affect (PA) and negative affect (NA). Instructions asked respondents to report their current mood state. It was translated into the Kazakh language. The PANAS was administered prior to and after performance of the ANT.

## 3 Results

Statistical analyses to address the aims of this article are reported, using the data reported by Kustubayeva et al. (2022). All analyses are new. Box-and-whisker plots for key variables are provided in Appendix 1 and confidence intervals for correlations are reported in Appendix 2. ANOVAs testing effects of flanker type, cue type, and stage of the task on RT and ERP amplitude are reported in Kustubayeva et al. (2022). Voltage plots and topographical maps to illustrate ERPS in different conditions may also be found in this publication.

### 3.1 Distributions of measures

Table 1 gives means for the RT and ERP amplitude measures at stages 1 and 3, together with Bonferroni-corrected *t*-tests for differences in means by stage. N100 amplitudes were multiplied by −1 as indicated previously. Mean values for the three network indices were consistent with those reported by Fan et al. (2002) and other investigations. The baseline RT data showed a substantial increase in RT over time (Hedges' *g* = 0.61), together with an increase in the alerting index (*g* = 0.53). However, the apparent improvement in alerting over time is misleading because of the baseline change. It reflects a smaller increase in RT in the double-cue condition (ΔRT = 14 ms) than in the no-cue condition (ΔRT = 28 ms; see Kustubayeva et al., 2022, Table 1). The Executive Control and Orienting indices did not change significantly over time. In the baseline condition, there were significant decreases over time in amplitudes of both N100 (*g* = 0.35) and P300 (*g* = 0.35). The only ERP network index measure to change was N100 alerting, which decreased (*g* = 0.28). In this instance, by contrast with RT data, amplitude decreased more in the double-cue condition (ΔN100 = –1.29 μV) than in the control condition (ΔN100 = −0.63 μV; see Kustubayeva et al., 2022, Table 2).

**Table 1:** Means (and SDs) for RT- and ERP-based indices at stages 1 and 3 of the task.

**Type of index**	**Baseline**			**Executive control**			**Alerting**			**Orienting**		
	**Stage 1**	**Stage 3**	***t***	**Stage 1**	**Stage 3**	***t***	**Stage 1**	**Stage 3**	***t***	**Stage 1**	**Stage 3**	***t***
RT	577 (74)	608 (82)	6.12^**^	113(44)	109 (40)	1.26	55 (24)	73 (31)	5.31^**^	37 (23)	44 (31)	2.15
N100	2.60 (1.90)	1.90 (1.83)	3.61^**^	0.14 (1.47)	0.15 (1.43)	1.35	1.73 (2.07)	1.08 (2.22)	2.88^**^	0.31 (1.53)	0.33 (1.57)	0.12
P300	4.24 (1.90)	3.76 (2.11)	3.33^**^	0.31 (1.48)	0.36 (1.25)	0.30	0.04 (1.69)	0.26 (2.03)	1.11	0.28 (1.39)	0.28 (1.23)	0.10

^**^p < 0.01.

**Table 2:** Intercorrelations of ERP-based indices at stages 1 and 3 of the task for N100.

		**Stage 1**				**Stage 3**			
		**Baseline**	**Executive control**	**Alerting**	**Orienting**	**Baseline**	**Executive control**	**Alerting**	**Orienting**
Stage 1	Baseline	–							
	Executive control	−0.149	–						
	Alerting	−0.094	0.069	–					
	Orienting	−0.045	0.236^*^	0.068	–				
Stage 3	Baseline	0.542^**^	−0.180	0.014	−0.031	–			
	Executive control	0.180	−0.184	0.045	0.005	0.016	–		
	Alerting	0.000	0.022	0.433^**^	−0.190	0.040	0.127	–	
	Orienting	−0.121	−0.109	−0.044	0.103	−0.015	−0.106	−0.225^*^	–

^*^p < 0.05, ^**^p < 0.01.

### 3.2 Stability of individual differences

Table 3 shows correlations between RT-based indices at stages 1 and 3. Baseline RT and executive control showed substantial stability over time, whereas alerting was less stable, and the two orienting measures were not significantly correlated. The table also confirms the independence of the three network indices at both time points. However, baseline RT was significantly positively correlated with alerting, and, to a lesser degree, with executive control.

**Table 3:** Intercorrelations of RT-based indices at stages 1 and 3 of the task.

		**Stage 1**				**Stage 3**			
		**Baseline RT**	**Executive control**	**Alerting**	**Orienting**	**Baseline RT**	**Executive control**	**Alerting**	**Orienting**
Stage 1	Baseline RT	–							
	Executive control	0.196^*^	–						
	Alerting	0.400^**^	−0.059	–					
	Orienting	0.168	0.159	0.042	–				
Stage 3	Baseline RT	0.796^**^	0.190	0.342^**^	0.093	–			
	Executive control	0.227^*^	0.650^**^	0.071	0.053	0.204^*^	–		
	Alerting	0.230^*^	0.002	0.305^**^	0.102	0.476^**^	0.007	–	
	Orienting	0.058	−0.023	0.152	0.170	0.080	0.107	0.192	–

^*^p < 0.05, ^**^p < 0.01.

Tables 2, 4 show the corresponding correlations between indices for ERP amplitude measures. Baseline RT and alerting showed the highest levels of stability, for both waves. For executive control, there was a significant cross-stage correlation only for P300, and orienting measures were unrelated. There were some small but significant correlations between metrics for different networks and baseline P300 amplitude was significantly negatively correlated with the alerting metric at both time points.

**Table 4:** Intercorrelations of ERP-based indices at stages 1 and 3 of the task for P300.

		**Stage 1**				**Stage 3**			
		**Baseline**	**Executive control**	**Alerting**	**Orienting**	**Baseline**	**Executive control**	**Alerting**	**Orienting**
Stage 1	Baseline	–							
	Executive control	0.141	–						
	Alerting	−0.247^*^	0.242^*^	–					
	Orienting	−0.196^*^	0.107	0.031	–				
Stage 3	Baseline	0.743^**^	0.036	−0.072	−0.294^**^	–			
	Executive control	−0.06	0.280^**^	0.067	−0.059	−0.015	–		
	Alerting	−0.050	0.117	0.422^*^	−0.004	−0.543^**^	0.013	–	
	Orienting	−0.760	−0.170	−0.129	0.073	−0.098	−0.080	−0.050	–

^*^p < 0.05, ^**^p < 0.01.

### 3.3 Inter-relationships of behavioral and ERP metrics

Table 5 shows correlations of the corresponding network indices derived from RTs, N100 amplitudes and P300 amplitudes, e.g., in the first cell, baseline N100 amplitude vs. baseline RT. The behavioral, RT-based measures were largely independent of the amplitude measures, with the exception of a small but significant correlation between N100 and baseline RT at stage 1. The table also shows a substantial negative correlation between the N100 and P300 executive control indices at stages 1 and 3, and a smaller positive association between baseline ERP amplitudes at stage 1.

**Table 5:** Correlations of corresponding network indices at stages 1 and 3 of the task.

	**Stage 1**				**Stage 3**			
	**Baseline**	**Executive control**	**Alerting**	**Orienting**	**Baseline**	**Executive control**	**Alerting**	**Orienting**
N100 vs. RT-based	−0.199^*^	0.047	−0.048	−0.079	0.026	−0.016	−0.060	0.067
P300 vs. RT-based	−0.060	0.086	0.049	0.116	−0.037	−0.170	0.126	−0.049
N100 vs. P300	0.236^*^	−0.478^**^	−0.095	−0.084	0.157	−0.388^**^	0.064	−0.011

^*^p < 0.05, ^**^p < 0.01.

### 3.4 Inter-relationships of initial index scores and change scores

Simple change scores (deltas) were calculated as (stage 3 index–stage 1 index) for behavioral and EEG indices, using the means given in Table 1. Table 6 shows correlations between stage 1 means for the four ANT indices and deltas. The table shows a consistent pattern of negative correlations, except for baseline RT which did not predict change in RT. Individuals with high baseline ERP amplitudes showed decreases on both baseline ERP indices, especially for N100. All three stage 1 index scores were negatively associated with the corresponding deltas. Notionally, these findings suggest that individuals initially poor at executive control improve in control over time, and those with good alerting and orienting deteriorate in these functions. However, these data are also consistent with the LIV which may reflect purely statistical factors such as regression to the mean (Burt and Obradović, 2013).

**Table 6:** Correlations between stage 1 scores for ANT indices and change scores (deltas).

**Delta**	**Stage 1 scores**		
	**Baseline**	**Executive control**	**Alerting**	**Orienting**
RT-based	−0.165	−0.507^**^	−0.455^**^	−0.514^**^
N100	−0.512^**^	−0.777^**^	−0.486^**^	−0.714^**^
P300	−0.231^*^	−0.684^**^	−0.413^*^	−0.728^**^

^*^p < 0.05, ^**^p < 0.01.

### 3.5 Correlations between change metrics

Residualized change measures were obtained by regressing the stage 3 index on the stage 1 index and saving the standardized residual. Residualized change scores correlate at zero with the stage 1 index. Table 7 shows intercorrelations of the two versions of the RT-based change scores, simple deltas and residuals. As expected, correlations for the two types of score were highly correlated for corresponding measures. For baseline RT, the correlation approached unity, but correlations were somewhat lower for network indices. The table also shows that change scores for alerting were substantially correlated with change scores for baseline RT.

**Table 7:** Intercorrelations of two types of change score measure for RT-based indices.

		**Deltas**				**Residuals**			
		**Base**.	**Exec. control**	**Alerting**	**Orient**.	**Base**.	**Exec. control**	**Alerting**	**Orient**.
Deltas	Base.	–							
	Exec. control	−0.028	–						
	Alerting	0.429^**^	−0.109	–					
	Orient.	0.102	0.222^*^	0.015	–				
Res.	Base.	0.986^**^	−0.026	0.422^**^	0.093	–			
	Exec. control	−0.018	0.862^**^	−0.099	0.184	0.003	–		
	Alerting	0.469^**^	−0.043	0.891^**^	0.071	0.494^**^	−0.038	–	
	Orient.	0.062	0.176	0.057	0.858^**^	0.068	0.174	0.139	–

Base., baseline; Exec. control, executive control; Orient., orienting; Res., residuals; ^*^p < 0.05, ^**^p < 0.01.

Tables 8, 9 show the correlations between change scores for the ERP measures. The two alternate change score measures were highly correlated for corresponding indices, but correlation magnitudes tended to be smaller than for RT, especially for executive control and orienting. Baseline and alerting change scores were strongly negatively correlated for P300 but not for N100.

**Table 8:** Intercorrelations of two types of change score measure for N100.

		**Deltas**				**Residuals**			

		**Base**.	**Exec. control**	**Alerting**	**Orient**.	**Base**.	**Exec. control**	**Alerting**	**Orient**.
Deltas	Base.	–							
	Exec. control	−0.096	–						
	Alerting	−0.063	0.079	–					
	Orient.	0.067	0.123	−0.008	–				
Res.	Base.	0.859^**^	0.015	−0.023	0.048	–			
	Exec. control	−0.183	0.630^**^	0.075	−0.128	−0.121	–		
	Alerting	−0.009	0.082	874^**^	−0.056	0.007	0.119	–	
	Orient.	0.112	0.020	−0.164	0.700^**^	0.061	−0.134	−0.216^*^	–

Base., baseline; Exec. control, executive control; Orient., orienting; Res., residuals; ^*^p < 0.05, ^**^p < 0.01.

**Table 9:** Intercorrelations of two types of change score measure for P300.

		**Deltas**				**Residuals**			

		**Base**.	**Exec. control**	**Alerting**	**Orient**.	**Base**.	**Exec. control**	**Alerting**	**Orient**.
Deltas	Base.	–							
	Exec. control	0.163	–						
	Alerting	−0.658^**^	0.044	–					
	Orient.	0.192	0.172	0.063	–				
Res.	Base.	0.973^**^	0.126	−0.663^**^	0.205^*^	–			
	Exec. control	0.098	0.729^**^	−0.020	0.049	0.076	–		
	Alerting	−0.689^**^	−0.028	0.991^**^	0.018	−0.720^**^	−0.022	–	
	Orient.	0.100	0.103	0.061	0.686^**^	0.065	−0.027	0.007	–

Base., baseline; Exec. control, executive control; Orient., orienting; Res., residuals; ^*^p < 0.05, ^**^p < 0.01.

We also investigated correlations between RT-based and ERP-based metrics for change. Significant correlations between behavioral and ERP metrics barely exceeded chance levels; the correlation matrix is provided in Appendix 3. However, N100 and P300 change scores were more extensively correlated. The first four columns of Table 10 show correlations between delta scores and the remaining columns show correlations between residuals. For corresponding measures, significant negative correlations between amplitudes were found for all three network index change scores. For example, individuals who showed increasing N100 executive control scores over time also showed decreasing P300 scores for both types of change score. Baseline P300 change was positively associated with N100 alerting change on both change measures. Additional correlations between N100 executive control change and other P300 measures were significant only for the delta change measure.

**Table 10:** Intercorrelations of N100 and P300 change scores, for two types of change score measure.

**N100**	**P300**							

	**Deltas**				**Residuals**			
	**Baseline**	**Executive control**	**Alerting**	**Orienting**	**Baseline**	**Executive control**	**Alerting**	**Orienting**
Baseline	0.096	0.172	−0.046	−0.008	0.113	0.097	−0.032	−0.142
Executive control	−0.306^**^	−0.464^**^	0.049	−0.321^**^	−0.166	−0.428^**^	0.037	−0.049
Alerting	0.287^**^	−0.136	−0.530^**^	0.157	0.384^**^	−0.020	−0.669^**^	−0.013
Orienting	−0.042	−0.141	0.080	−0.217^*^	−0.015	0.047	0.191	−0.273^**^

^*^p < 0.05, ^**^p < 0.01.

### 3.6 Correlations with individual differences in affect

Comparisons of pre- and post-task means on the PANAS showed a significant decrease in NA during the task, *t*_(101)_ = 2.66, *p* < 0.01. Means (and SDs) were 16.8 (4.4) pre-task and 15.7 (3.9) post-task. PA also decreased over time, *t*_(101)_ = 2.67, *p* < 0.01. Means (and SDs) were 29.4 (5.8) pre-task and 27.9 (7.1) post-task. Thus, performing the task appeared to produce a general decline in emotional arousal, but was not otherwise stressful. There were significant test-retest correlations for both NA (*r* = 0.431, *p* < 0.001) and for PA (*r* = 0.676, *p* < 0.001). Subsequent analyses used measures of NA and PA averaged across pre- and post-task administrations on this basis.

Correlations between the two affect scales and RT-based measures are shown in Table 11. The residualized measures were used to index change. At stage 1, higher PA was associated with faster baseline RT and with lower values for the Alerting index. NA was positively correlated with the Executive Control index, i.e., poorer control. However, neither affect dimension was significantly correlated with any of the change scores. We also correlated PA and NA with N100 and P300 amplitude measures (stage 1 and residualized change scores), but significant correlations did not exceed chance levels.

**Table 11:** Correlations between RT-based indices and affect scales at stage 1 and for residualized (deltas).

	**Stage 1**				**Residuals**			

	**Baseline**	**Executive control**	**Alerting**	**Orienting**	**Baseline**	**Executive control**	**Alerting**	**Orienting**
PA	−0.281^**^	−0.105	−0.239^*^	−0.011	−0.035	−0.085	−0.060	−0.176
NA	0.132	0.259^**^	0.031	0.136	−0.026	−0.039	0.014	−0.127

^*^p < 0.05, ^**^p < 0.01.

## 4 Discussion

The current study explored individual differences in behavioral and electroencephalographic metrics provided by the ANT (Fan et al., 2002), including their suitability as markers for sustained attention. Table 12 summarizes the outcomes of the data analyses we performed. Findings differed from expectations in several respects. We confirmed that the majority of metrics were stable over the course of the experiment but level of stability varied considerably. Contrary to expectation, RT-based and ERP amplitude metrics were not significantly correlated, in general, raising questions about which provides the more valid indices of attentional network functioning. Analyses of simple change scores (deltas) showed that almost all were subject to the LIV (Wilder, 1957); that is, initial scores did not identify impairments in attentional network functioning that deepened over time. Data also showed moderate divergence of delta and residualized scores for some measures. Finally, positive and negative affect both predicted behavioral metrics but not ERP amplitudes or change metrics.

**Table 12:** Summary of tests of hypotheses.

**Research issue**	**Hypothesis**	**Outcomes**
Stability of individual differences	RT and ERP metrics are stable over time	Stabilities vary with metrics - Highest for baseline and RT metrics - Lowest for orienting
Inter-relationships of RT and ERP metrics	Corresponding metrics are correlated	RT and ERP metrics were largely independent N100 and P300 executive control indices were negatively correlated
Inter-relationships of initial and change scores.	Differing predictions from impairment model and LIV	LIV was supported for both RT and ERP metrics
Correlations between change metrics	Exploratory	Deltas and residualized measures were substantially correlated, but dependent on measure Changes in baseline and Alerting were correlated for RT and P300 metrics
Correlations with affect	NA is associated with poorer executive control and better orienting	NA related to poorer executive control PA related to faster baseline RT and weaker alerting Affect was unrelated to change scores and to ERPs

Next, we consider the implications of these findings for three focal issues. First, we discuss choice of metrics for assessing individual differences in sustained attention, including behavioral vs. electroencephalographic measures, and network indices vs. baseline measures. Second, we address the prediction of change in sustained attention; how can we identify individuals vulnerable to performance decrement? Third, we compare our findings on associations between affect and attentional performance with results of previous studies.

### 4.1 Metrics for individual differences in sustained attention

Traditionally, sustained attention research has utilized a variety of behavioral metrics, such as the hit and false alarm rate measures commonly used in vigilance research (See et al., 1995). Such metrics can be used to index both overall level of performance on a task requiring sustained attention as well as temporal decrement (Davies and Parasuraman, 1982). Cognitive neuroscience research suggests two innovations. First, the identification of multiple brain networks for attention (Petersen and Posner, 2012) implies that we can discriminate multiple metrics for network functioning that provide a more informative specification of stability and change in sustained attention than overall performance measures. Second, psychophysiological measures of network function such as ERP amplitudes may provide more valid assessment of networks than behavioral data. The present findings suggest some challenges in deriving sustained attention metrics via the cognitive neuroscience approach, as we next discuss. The temporal stability of metrics varies across the different metrics, behavioral and ERP metrics fail to converge, and metrics for alerting are confounded with baseline response.

Much of the applied literature assumes that individual differences in network function in part reflect stable, traitlike attributes of the brain. For example, research on depression (Sinha et al., 2022) and schizophrenia (Spagna et al., 2018) has assumed that deficits in executive function are integral to the clinical condition concerned. Studies of test-retest reliability (stability) of the Fan et al. (2002) behavioral indices across test sessions have shown that stability is higher for executive control than for alerting and orienting (Fan et al., 2001; Kong et al., 2024). Variation in metric stability across networks may reflect both actual network stability and variation in reliability of measurement (MacLeod et al., 2010). The present study is the first to examine stability within a prolonged test session. Similar to cross-session analyses (Kong et al., 2024), we found greater temporal stability for executive control than for alerting and orienting. We also found that baseline RT stability was more stable than any of the derived indices.

A contrasting picture emerged from the ERP data, in which alerting showed the highest stability from stage 1 to stage 3, although the correlation magnitudes for the two waves were modest. Executive control showed significant temporal stability for P300 but not N100, which may reflect that the later waveform represents the primary expression of network activity (Neuhaus et al., 2010). The orienting ERP metric showed no stability. As for RT metrics, the highest stability was found for ERPs in the baseline condition.

We also anticipated convergence between behavioral and ERP metrics, given that they reflect the same underlying construct of network activity. However, both N100 and P300 amplitudes were largely independent of the corresponding behavioral metrics, with the exception of a small but significant association between N100 and speed of response in the baseline condition. We also found an unanticipated negative correlation between N100 and P300 amplitudes for executive control, perhaps reflecting individual differences in strategy for resource allocation across processing stages. The effects of cue and flanker type in the dataset (see Kustubayeva et al., 2022) were consistent with previous behavioral (Fan et al., 2002) and ERP (Kaufman et al., 2016; Neuhaus et al., 2010) findings, supporting the validity of both types of metric at the level of group data. However, at the individual level, RTs and amplitude measures do not seem to be measuring the same construct.

A final measurement challenge is the role of baseline response in assessment of sustained attention. In the behavioral data, we found that baseline RT was significantly positively correlated with the alerting index at both time points. That is, individuals who were slow to respond scored higher on alerting. The association might reflect slow-baseline individuals being more dependent on exogenous cuing or a statistical effect such that individuals who are slow with no cue have more scope for speeding up when the alerting cue is present. In either case, there is a confound that complicates interpretation of individual differences in alerting. The P300 alerting index was also similarly confounded with baseline P300 amplitude. The negative baseline—alerting correlation indicates that individuals with a smaller P300 response at baseline scored higher on alerting. The magnitude of the correlation increased over time from −0.247 at stage 1 to −0.543 at stage 3. Again, it is unclear that the index can be interpreted as a pure network activity measure at the individual level.

### 4.2 Predicting change in sustained attention

The prediction of individual differences in temporal change is important both theoretically and practically. From a theoretical perspective, cognitive neuroscience accounts of sustained attention (e.g., Langner and Eickhoff, 2013) should identify attributes of network functioning that prefigure declines in network functioning. From an applied standpoint, temporal decline in sustained attention threatens performance and safety in a variety of real-world contexts including transportation, industrial operations and medical monitoring (Warm et al., 2008), requiring methods for identifying operators at risk of temporal decrement. The impairment model of decrement suggests a possible strategy for predicting change, i.e., individuals in whom the network is already impaired should be at most risk of decrement as time progresses. The contrary view is that individual differences in change are governed by the LIV (Wilder, 1957), so that higher scores on an index will tend to regress to lower values over time.

The current data support the LIV rather than the impairment model. Table 5 shows that all three Fan et al. (2002) behavioral indices conformed to the LIV. Given the scoring of the ANT, individuals with poor executive control (high index scores) tended to improve over time, whereas high control individuals at stage 1 had poorer control at the later task stage. Alerting and orienting indices showed a similar pattern. Similarly, initial levels of the ERP-based network indices were negatively associated with change scores. Baseline measures showed the strongest tendency toward vigilance-like decrements in group-level mean data (Table 1). Mean RT was longer at stage 3 than stage 1, and both waves decreased in amplitude over time. We might expect that baseline measures would thus provide the strongest support for the impairment model. However, baseline measures showed a trend toward the LIV, though somewhat weaker than for the derived network indices. Baseline RT did not predict ΔRT, and baseline ERP amplitudes showed moderate LIV effects. The impairment model is quite well-supported in group-level data; e.g., factors that impair initial level of vigilance also tend to induce greater vigilance decrement, consistent with resource theory (See et al., 1995; Warm et al., 2008). Overall, the ANT indices do not seem to identify individuals at risk of progressively increasing impairment over time; regression to the mean is unlikely to be useful in applied contexts.

The delta score provides the simplest metric for change but other methods for indexing change have been proposed (Burt and Obradović, 2013). We compared deltas with residualized change scores that are independent of the initial value for the measure. Correlations between deltas and residuals varied from close to unity for baseline RT to values in the 0.6–0.7 range for certain of the ERP change scores. Especially for ERP data, the choice of change metric may make a difference to the correlation with external criteria. Behavioral and ERP metrics for change diverged, but there was unexpected interdependence between N100 and P300 metrics, with negative correlations for both alerting and executive control indices.

Change score data, similar to absolute level data, showed confounding between baseline and alerting indices (Tables 6, 7). Somewhat confusingly, the correlation between change in baseline scores and change in alerting index score was positive for RT, negative for P300 amplitude, and non-significant for N100. These correlations can be understood in the context of temporal change in the variables. RT, as previously noted, increased more over time in no-cue than in double-cue conditions. Thus, an individual whose baseline RT showed a large temporal increase, but whose double-cue RT did not change, would show a large but possibly artifactual increase on the RT alerting index. P300 response declined similarly in both no-cue and double-cue conditions so that there was no net effect on alerting, consistent with alerting primarily influencing N100 rather than P300 (Kustubayeva et al., 2022; Neuhaus et al., 2010). However, at the individual level, the substantial negative correlation implies that individuals whose baseline P300 increased the most also showed the smallest amplitude increases in double-cue conditions, reducing scores on the alerting index. As for absolute-level data, these findings suggest challenges for interpreting temporal change in the alerting index. Baseline—alerting correlation was similar for both deltas and residualized change scores, implying that the issue is not a consequence of confounding of baseline and change scores, which is eliminated by residualization. However, the N100 alerting change index, which appears to be one of the more promising metrics for vigilance decrement, was unconfounded by change in baseline N100 response, although, curiously, the index was negatively correlated with P300 change measures (Table 8).

Taken together, these findings show the difficulty of indexing change at an individual level. Behavioral and RT-based change metrics should converge but do not. Alerting and baseline change metrics should not converge, but they do for RT and P300 measures. There were also unexpected relationships between N100 and P300 changes. Using residualized change scores in place of simple deltas does not mitigate these issues.

### 4.3 Correlations with affect

The final aim of this study was to evaluate how choice of metric influenced correlations with affective state. We focused on affect because there is a fairly substantial literature on affective correlates of the ANT, utilizing both clinical and non-clinical samples (e.g., Matthews and Zeidner, 2012; Moriya, 2018; Pacheco-Unguetti et al., 2010; Sinha et al., 2022). Impacts of emotional stress on attention network functioning are also relevant to human factors applications such as predicting attentional impairment in vehicle driving (Guinosso et al., 2016). Data from some clinical groups show reasonable consistency across studies; for example, Sinha et al.'s (2022) meta-analysis of 11 studies confirmed that depressed patients have poorer executive control than healthy controls. However, correlational studies in non-clinical groups show some inconsistencies (e.g., Finucane et al., 2010; Moriya, 2018). These inconsistencies may in part reflect limited validity of the ANT indices at the individual level.

In the present study, we found a small but significant association between state negative affect and the behavioral executive control index (i.e., poorer control), consistent with other studies in clinical groups (Pacheco-Unguetti et al., 2010; Sinha et al., 2022) and a non-clinical sample (Matthews and Zeidner, 2012). In this instance, inconsistency across studies may be a product of the low effect size and need for statistical power. We replicated Noh et al.'s (2012) finding of a significant negative association between positive affect and alerting. However, we also found that positive affect was associated with faster baseline response, and baseline RT is confounded with the ANT index as previously discussed. This finding reinforces the need to examine baseline data as well as the computed indices (Finucane et al., 2010).

While behavioral data were broadly consistent with previous findings, we found no more than chance-level associations between affective state and ERP-based data, so that the neurological basis for the behavioral correlates of affect remains uncertain. We also anticipated that affect might be related to the change metrics, given that sustained attention deficits are found in a variety of clinical groups including depression and posttraumatic stress disorder (Fortenbaugh et al., 2018) and chronic fatigue patients (Fernández-Quirós et al., 2023). However, no significant associations between affective state and change metrics were found.

### 4.4 Limitations

One possible limitation is that ERP amplitude measures are not optimal for evaluating individual differences in brain network functioning. In support of ERP analysis, group-level analyses support the validity of amplitude measures (Kaufman et al., 2016; Kustubayeva et al., 2022; Neuhaus et al., 2010) and there is an extensive literature on individual-difference correlates of ERPs (e.g., Hajcak et al., 2010). However, there are various methodological challenges for research on individual differences in ERPs (e.g., Clayson et al., 2021b) and alternative EEG metrics might be more diagnostic for sustained attention. These include measures of coherence (Kamzanova et al., 2020) and functional connectivity (Imperatori et al., 2021), as well as those derived from deep learning EEG models (Kamrud et al., 2021). Other types of neurological measure such as fMRI (Kong et al., 2024) might also enhance validity of measurement.

We utilized the standard ANT here because of its basis in cognitive neuroscience theory (e.g., Fan et al., 2007) and the extensive research base supporting its usage (de Souza Almeida et al., 2021). The task produced moderate temporal declines in baseline RT and ERP response. Effect sizes were consistent with those previously reported in vigilance studies, according to a meta-analysis (See et al., 1995). However, “vigilance” tasks are heterogeneous and vary in their demands on information-processing (Warm et al., 2008), and the magnitude of temporal performance decrement varies markedly across different vigilance tasks (See et al., 1995). Correlational studies of multiple vigilance tasks have shown that while performance measures tend to correlate significantly across tasks, correlational magnitudes are often quite modest, falling into the 0.3–0.4 range (Matthews et al., 1993, 2017b). Recent research utilizing the ANTI-VEA has made a useful contribution to this literature in showing dissociation between executive and arousal vigilance indices. However, the field lacks a comprehensive dimensional model of performance encompassing the spectrum of vigilance tasks. Thus, the current results may not generalize to other vigilance tasks. Indeed, convergence between behavioral and electroencephalographic indices of decrement might be higher on vigilance tasks that produce larger-magnitude temporal changes. It would also be of interest to investigate inter-relationships of the two types of vigilance, executive and arousal vigilance described by Luna et al. (2022).

Statistical limitations include lack of power to detect small effect sizes, such as those found here between affect and ANT indices. There is also a risk of Type 1 error in reporting of significant correlations, given the number of analyses. We chose not to apply familywise corrections to significance levels because in some cases we report correlations between arithmetically dependent variables, e.g., initial score vs. delta, which existing correction procedures are not designed to address. We urge caution in interpreting correlations of 0.01 < *p* < 0.05. The key findings summarized in Table 12 are mostly supported by correlations that were significant at *p* < 0.01 or better. However, significant correlations between the ANT indices and affect were of modest magnitude, in the 0.2–0.3 range. These associations require replication although correlation magnitudes were in line with those found in previous studies (e.g., Matthews and Zeidner, 2012; Noh et al., 2012). Indexing temporal change solely through comparing early and late stages of performance may also be limiting.

A reviewer of this manuscript noted that using mean RT to index behavioral response may be questionable because RT distributions are commonly skewed. We used means here because this metric is specified in the Fan et al. (2002) formulae which are routinely used in ANT research. We also confirmed that reliability of RT measurement across different conditions was high, suggesting stability in measurement of individual differences in mean. However, the strong intercorrelation of RTs across different task conditions limits the accuracy of measurement of the Fan et al. (2002) difference scores, consistent with previous psychometric critiques of the ANT (MacLeod et al., 2010). Reliabilities of ERP-based difference scores are also sensitive to the intercorrelation of the constituent scores of the formula (Clayson et al., 2021a). Future research might look more closely at properties of intra-individual RT distributions. In the present data analysis, we checked inter-individual distributions of mean RT and we did not detect excessive skew. On a similar point, there are also concerns about the stability of peak ERP amplitude as an individual difference measure, and it has been recommended to use mean amplitude within a defined window as a better alternative (Clayson et al., 2021b; Luck et al., 2021). We chose to use peak amplitude for comparability with our companion article (Kustubayeva et al., 2022) and other ERP studies of the ANT (e.g., Neuhaus et al., 2010). However, it would be desirable for future research to compare psychometric properties of peak and mean amplitude, given the advantages of the latter.

### 4.5 Conclusion

The ANT has become established as a leading research tool for investigating multiple aspects of attention (de Souza Almeida et al., 2021), including sustained attention (Zholdassova et al., 2021). Group-level data from many studies support its usage for investigating a variety of research issues including clinical deficits in attention and attentional impairments in real-life settings. In the current data, the N100 alerting index showed promise as a metric for sustained attention. However, the present findings also suggest that researchers and practitioners should use considerable caution in using the ANT to assess and temporal performance deficits in individuals. Specifically, we identified the following issues:

- Metrics vary in stability; individual differences in temporal change largely reflect the LIV. Further work is necessary to identify neurocognitive measures that can be used in applied settings to identify vulnerability to temporal decrements in brain network functioning.
- Group-level data show that ERP-based metrics are informative about the neural processes supporting attentional performance. However, at the individual level, behavioral and ERP-based metrics of network functioning failed to converge, both for absolute-level and change metrics. Measuring the individual's network efficiency appears to be challenging, and novel assessment methods may be necessary.
- The behavioral alerting index tended to be correlated with baseline RT. Group-level data suggested participants become increasingly dependent on the alerting cue over time. Similarly, interpreting individual-level data requires attention to changes in baseline as well as changes in the alerting index.
- Correlations between affect measures and the ANT were broadly consistent with previous findings. However, findings also show the need to examine baseline RT correlates as well as those of the network indices.

Several authors (e.g., de Souza Almeida et al., 2021; MacLeod et al., 2010) have drawn attention to psychometric limitations of the ANT including its dependence on difference-score indices. The current data are consistent with these critiques and highlight the need to examine ANT data in depth rather than relying on analyses of the three Fan et al. (2002) indices alone.

## Data Availability

The datasets presented in this article are not readily available because restrictions apply to the datasets. Requests to access the datasets should be directed to almira.kustubaeva@kaznu.edu.kz.
