# Supplementary material for: Individual differences in brain attention networks: the challenge of indexing temporal change
Source: Front Cognit. 2025 Jun 18;4:1547773. doi: 10.3389/fcogn.2025.1547773 (PMC13281110; doi:10.3389/fcogn.2025.1547773)

**Appendix 1. Box and Whisker Plots**

**Table A1.1.** Baseline RT and ANT index values at stage 1 (S-1) and stage 3 (S-3).


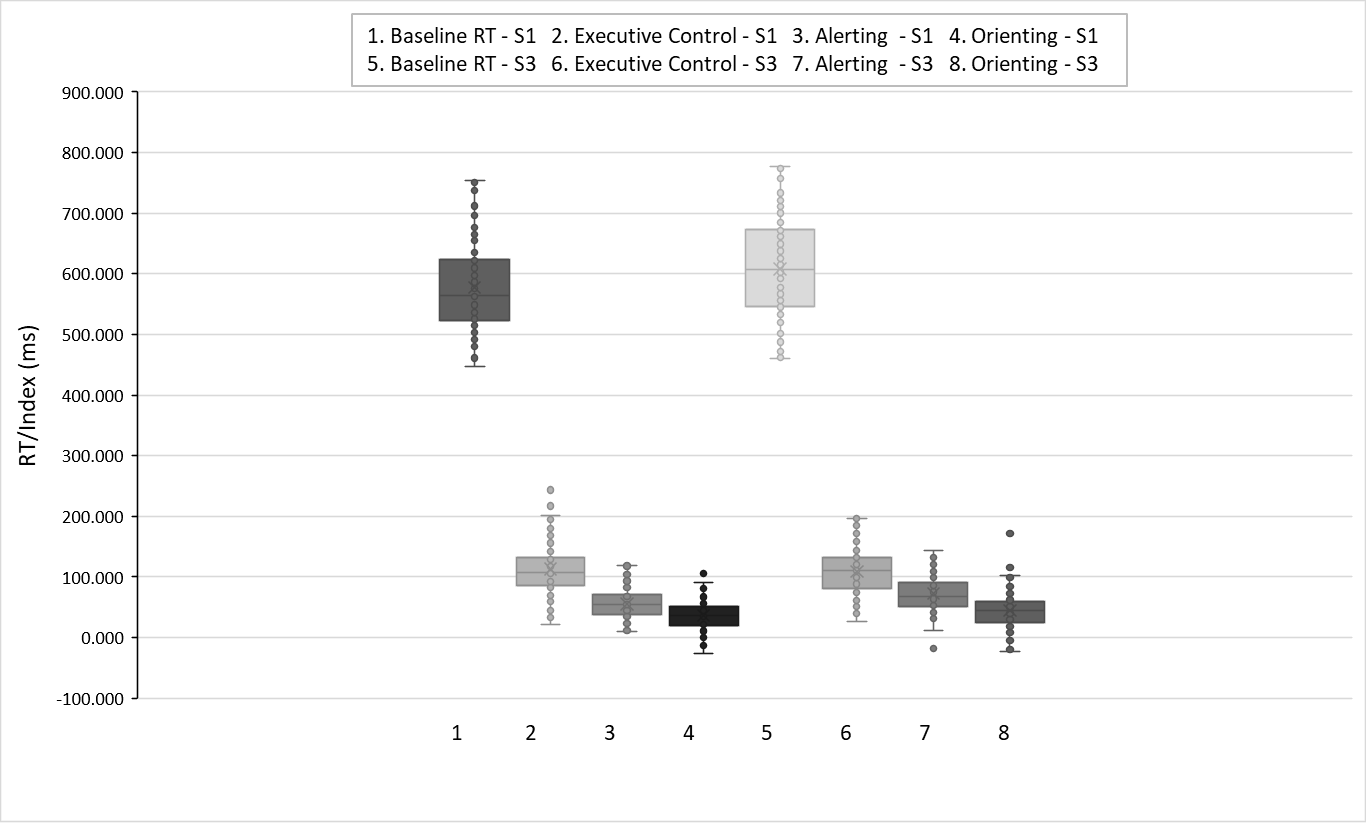


**Table A1.2.** Baseline N100 amplitude and N100 network index values at stage 1 (S-1) and stage 3 (S-3).


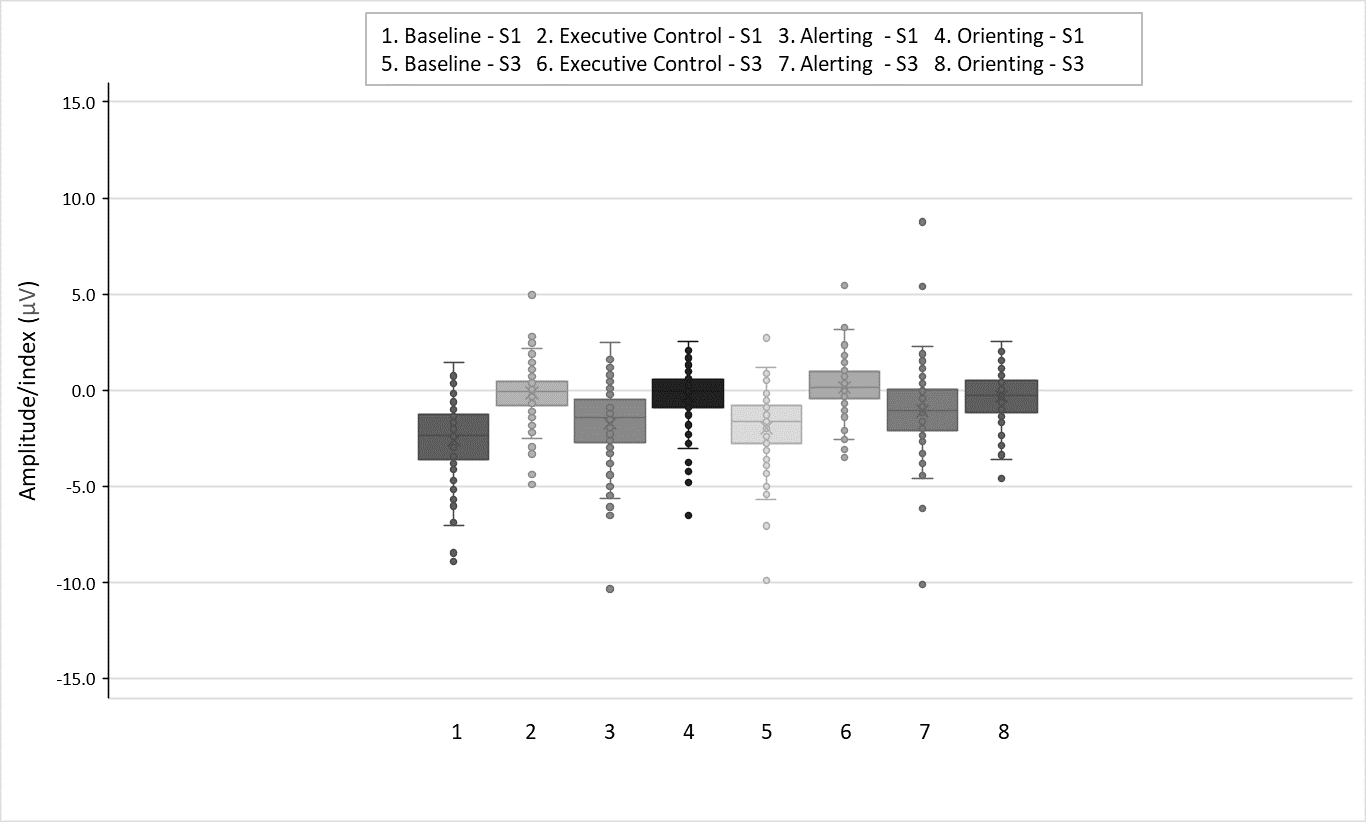


**Table A1.3.** Baseline P300 amplitude and P300 network index values at stage 1 (S-1) and stage 3 (S-3).


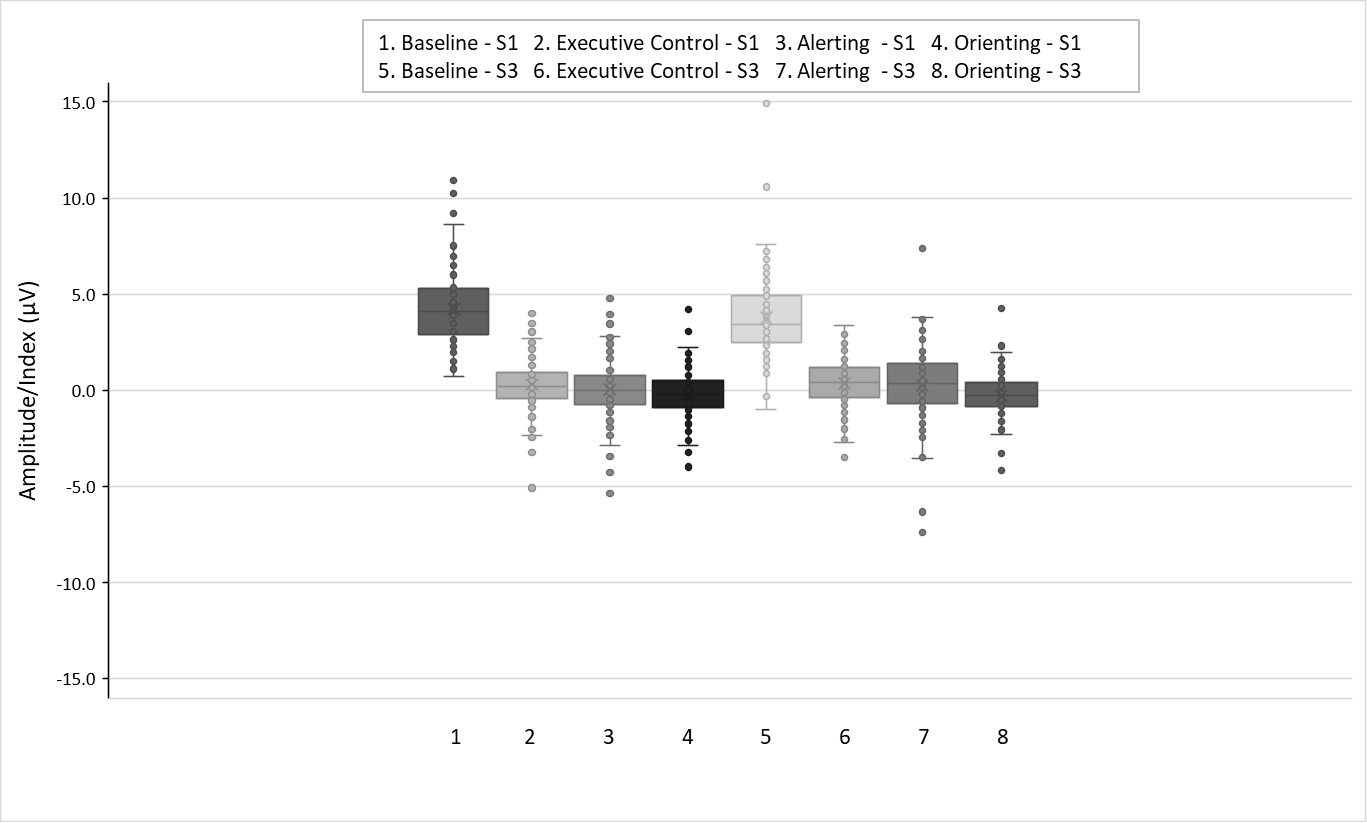


**Table A1.4.** Change scores (deltas) for baseline RT and ANT index values.


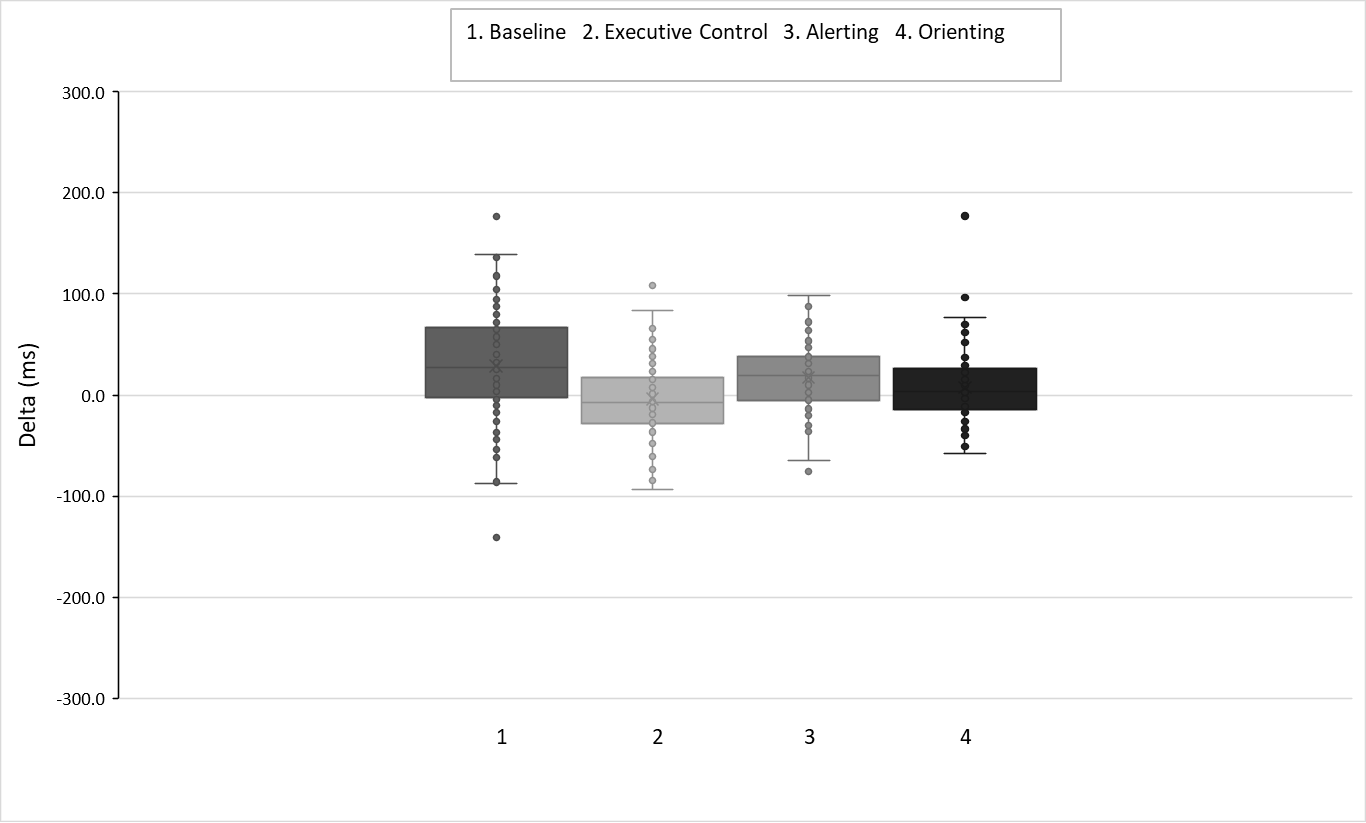


**Table A1.5.** Change scores (deltas) for baseline N100 amplitude, N100 network index values, baseline P300 amplitude, and P300 network index values.


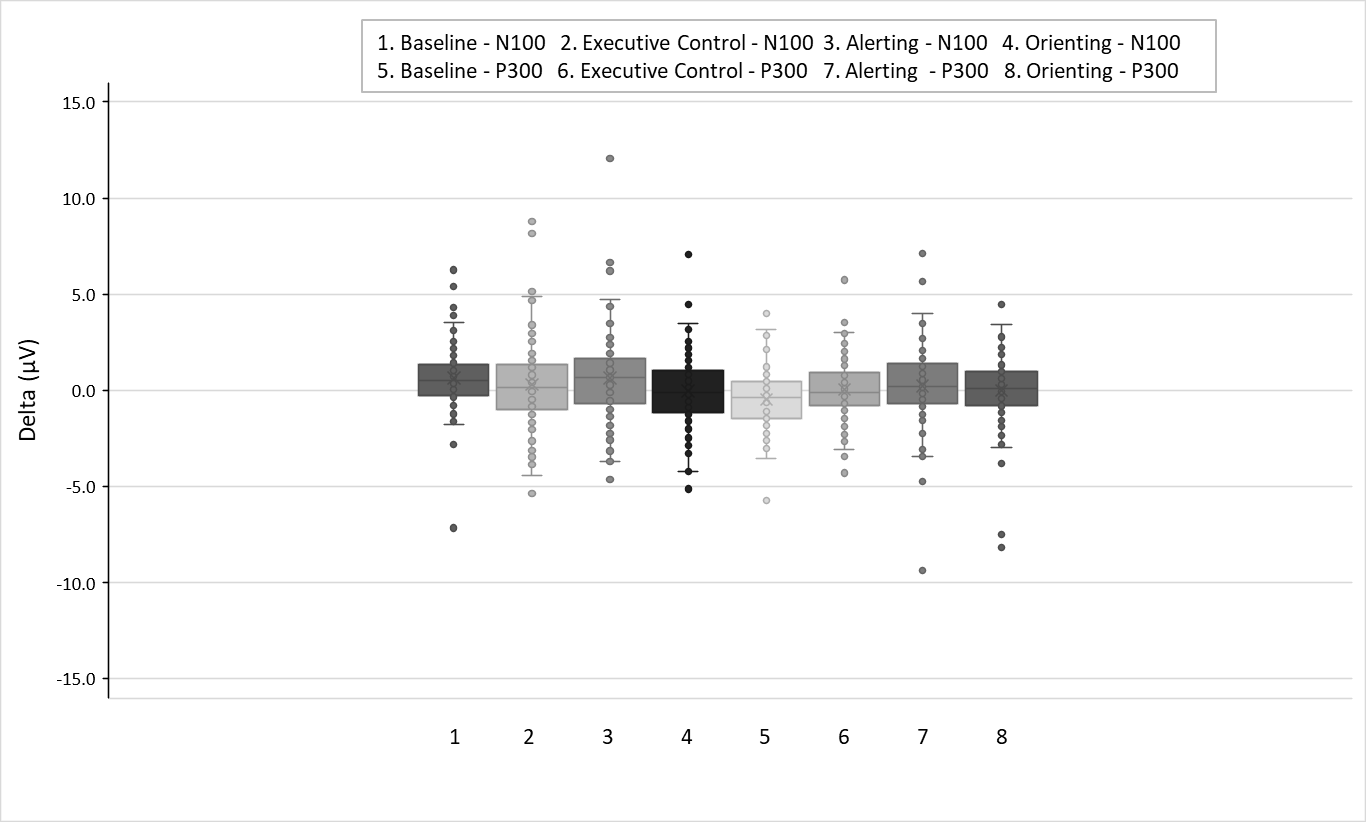


**Table A1.6.** Change scores (residuals) for baseline RT and ANT network index values.


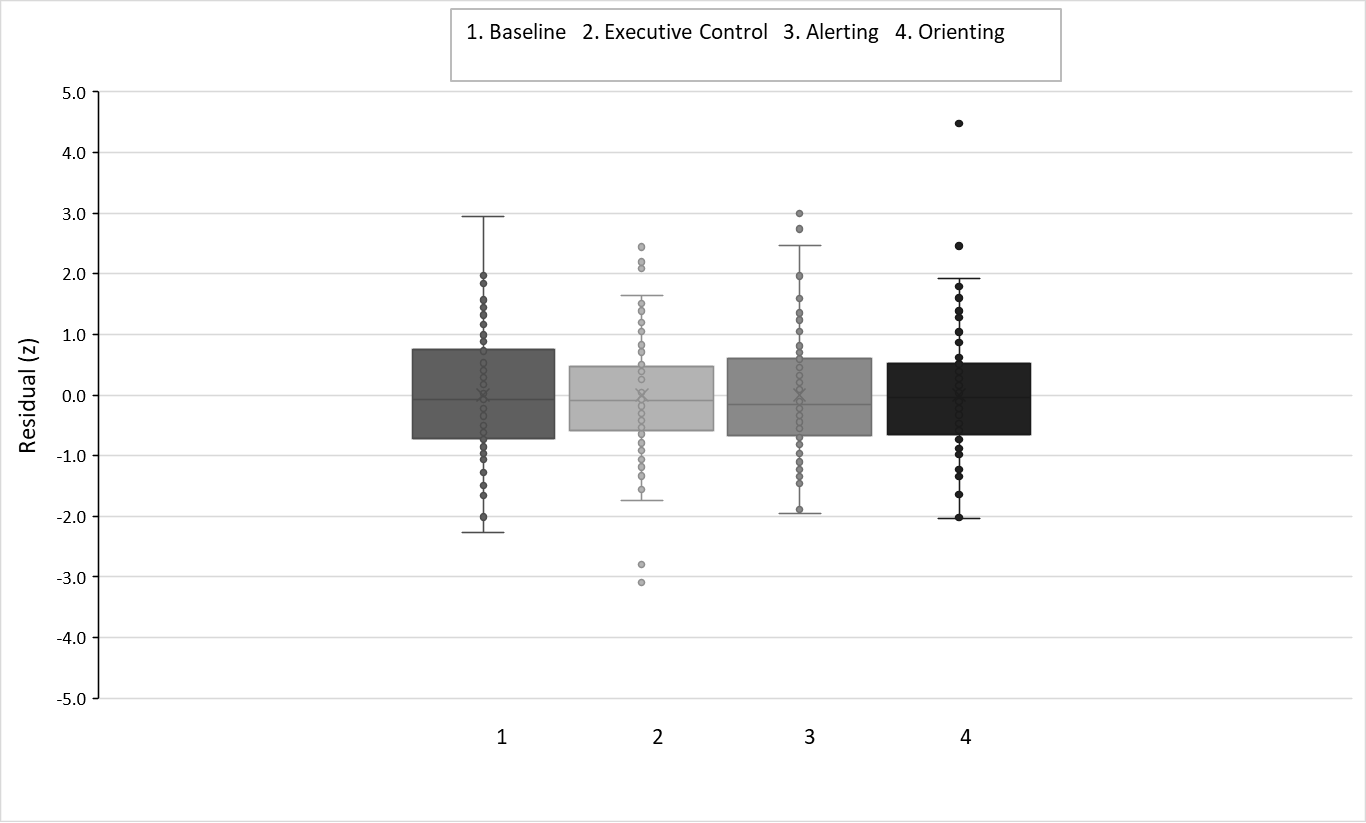


**Table A1.7.** Change scores (residuals) for baseline N100 amplitude, N100 network index values, baseline P300 amplitude, and P300 network index values.


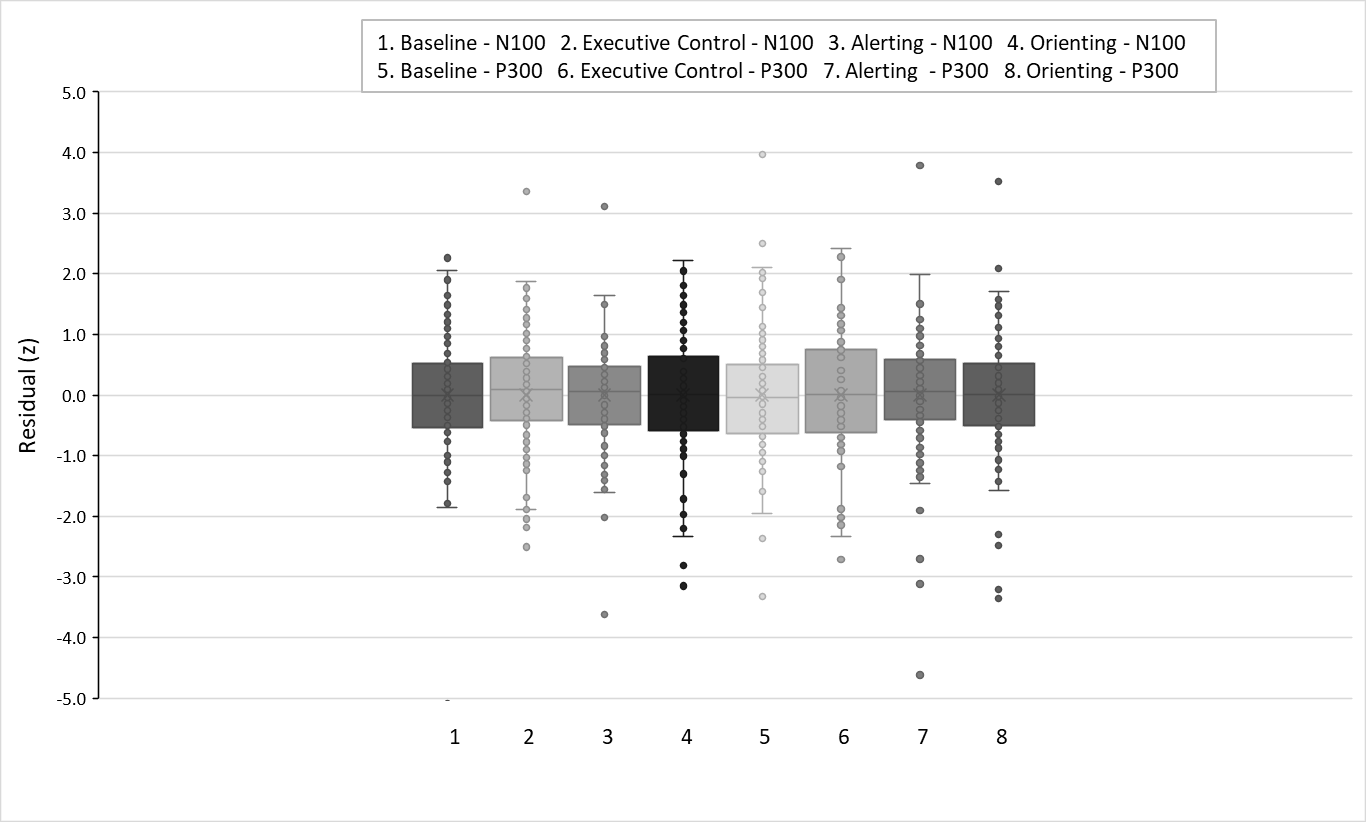

Supplement: Supplementary file 1 [file Table_1.docx]
