# Supplementary material for: Individual differences in brain attention networks: the challenge of indexing temporal change
Source: Front Cognit. 2025 Jun 18;4:1547773. doi: 10.3389/fcogn.2025.1547773 (PMC13281110; doi:10.3389/fcogn.2025.1547773)
Supplement: Supplementary file 2 [file Table_2.docx]

**Appendix 2. Confidence intervals (CIs) for correlations.**

**Table A2.1.** Intercorrelations of RT-based indices at stages 1 and 3 of the task: 95% CIs.

|  | | | Stage 1 | | | | Stage 3 | | | |
| --- | --- | --- | --- | --- | --- | --- | --- | --- | --- | --- |
|  | | | Baseline RT | Executive Control | Alerting | Orienting | Baseline RT | Executive Control | Alerting | Orienting |
| Stage 1 | Baseline RT | Lower |  |  |  |  |  |  |  |  |
|  |  | Upper |  |  |  |  |  |  |  |  |
|  | Executive Control | Lower | -0.017 |  |  |  |  |  |  |  |
|  |  | Upper | 0.382 |  |  |  |  |  |  |  |
|  | Alerting | Lower | 0.184 | -0.261 |  |  |  |  |  |  |
|  |  | Upper | 0.573 | 0.123 |  |  |  |  |  |  |
|  | Orienting | Lower | -0.060 | -0.009 | -0.198 |  |  |  |  |  |
|  |  | Upper | 0.392 | 0.336 | 0.261 |  |  |  |  |  |
| Stage 3 | Baseline RT | Lower | 0.724 | -0.003 | 0.116 | -0.103 |  |  |  |  |
|  |  | Upper | 0.853 | 0.353 | 0.521 | 0.298 |  |  |  |  |
|  | Executive Control | Lower | -0.002 | 0.506 | -0.137 | -0.144 | -0.013 |  |  |  |
|  |  | Upper | 0.424 | 0.772 | 0.264 | 0.263 | 0.395 |  |  |  |
|  | Alerting | Lower | 0.046 | -0.248 | 0.164 | -0.083 | 0.324 | -0.228 |  |  |
|  |  | Upper | 0.415 | 0.250 | 0.437 | 0.258 | 0.617 | 0.234 |  |  |
|  | Orienting | Lower | -0.192 | -0.220 | -0.133 | -0.048 | -0.138 | -0.117 | 0.016 |  |
|  |  | Upper | 0.293 | 0.200 | 0.400 | 0.401 | 0.282 | 0.323 | 0.338 |  |

*Note.* In this and subsequent tables, 95% CIs were computed using the SPSS 29.0 Bootstrap procedure with 1000 samples and case re-sampling with replacement.

**Table A2.2**. Intercorrelations of ERP-based indices at stages 1 and 3 of the task for N100: 95% CIs.

|  | | | Stage 1 | | | | Stage 3 | | | |
| --- | --- | --- | --- | --- | --- | --- | --- | --- | --- | --- |
|  | | | Baseline RT | Executive Control | Alerting | Orienting | Baseline RT | Executive Control | Alerting | Orienting |
| Stage 1 | Baseline RT | Lower |  |  |  |  |  |  |  |  |
|  |  | Upper |  |  |  |  |  |  |  |  |
|  | Executive Control | Lower | -0.323 |  |  |  |  |  |  |  |
|  |  | Upper | 0.048 |  |  |  |  |  |  |  |
|  | Alerting | Lower | -0.292 | -0.187 |  |  |  |  |  |  |
|  |  | Upper | 0.133 | 0.352 |  |  |  |  |  |  |
|  | Orienting | Lower | -0.287 | -0.003 | -0.224 |  |  |  |  |  |
|  |  | Upper | 0.181 | 0.452 | 0.348 |  |  |  |  |  |
| Stage 3 | Baseline RT | Lower | 0.366 | -0.363 | -0.213 | -0.236 |  |  |  |  |
|  |  | Upper | 0.720 | 0.006 | 0.227 | 0.174 |  |  |  |  |
|  | Executive Control | Lower | -0.017 | -0.413 | -0.222 | -0.226 | -0.172 |  |  |  |
|  |  | Upper | 0.360 | 0.071 | 0.258 | 0.210 | 0.214 |  |  |  |
|  | Alerting | Lower | -0.180 | -0.300 | 0.192 | -0.353 | -0.210 | -0.045 |  |  |
|  |  | Upper | 0.206 | 0.333 | 0.636 | 0.155 | 0.307 | 0.318 |  |  |
|  | Orienting | Lower | -0.290 | -0.322 | -0.220 | -0.086 | -0.224 | -0.335 | -0.416 |  |
|  |  | Upper | 0.047 | 0.136 | 0.138 | 0.320 | 0.182 | 0.175 | 0.001 |  |

**Table A2.3**. Intercorrelations of ERP-based indices at stages 1 and 3 of the task for P300: 95% CIs.

|  | | | Stage 1 | | | | Stage 3 | | | |
| --- | --- | --- | --- | --- | --- | --- | --- | --- | --- | --- |
|  | | | Baseline RT | Executive Control | Alerting | Orienting | Baseline RT | Executive Control | Alerting | Orienting |
| Stage 1 | Baseline RT | Lower |  |  |  |  |  |  |  |  |
|  |  | Upper |  |  |  |  |  |  |  |  |
|  | Executive Control | Lower | -0.079 |  |  |  |  |  |  |  |
|  |  | Upper | 0.350 |  |  |  |  |  |  |  |
|  | Alerting | Lower | -0.457 | -0.020 |  |  |  |  |  |  |
|  |  | Upper | -0.011 | 0.478 |  |  |  |  |  |  |
|  | Orienting | Lower | -0.430 | -0.179 | -0.178 |  |  |  |  |  |
|  |  | Upper | 0.076 | 0.360 | 0.255 |  |  |  |  |  |
| Stage 3 | Baseline RT | Lower | 0.603 | -0.130 | -0.364 | -0.514 |  |  |  |  |
|  |  | Upper | 0.833 | 0.230 | 0.059 | -0.021 |  |  |  |  |
|  | Executive Control | Lower | -0.242 | 0.084 | -0.120 | -0.270 | -0.165 |  |  |  |
|  |  | Upper | 0.122 | 0.445 | 0.261 | 0.145 | 0.130 |  |  |  |
|  | Alerting | Lower | -0.423 | -0.171 | 0.182 | -0.369 | -0.708 | -0.163 |  |  |
|  |  | Upper | 0.170 | 0.400 | 0.634 | 0.332 | -0.279 | 0.184 |  |  |
|  | Orienting | Lower | -0.394 | -0.419 | -0.317 | -0.262 | -0.296 | -0.307 | -0.361 |  |
|  |  | Upper | 0.046 | 0.116 | 0.085 | 0.432 | 0.085 | 0.160 | 0.273 |  |

**Table A2.4.** Correlations of corresponding network indices at stages 1 and 3 of the task: 95% CIs.

|  |  | Stage 1 | | | | Stage 3 | | | |
| --- | --- | --- | --- | --- | --- | --- | --- | --- | --- |
|  |  | Baseline | Executive Control | Alerting | Orienting | Baseline | Executive Control | Alerting | Orienting |
| N100 vs. RT-based  P300 vs. RT-based | Lower | 0.007 | -0.265 | -0.195 | -0.131 | -0.289 | -0.199 | -0.118 | -0.278 |
|  | Upper | 0.376 | 0.171 | 0.275 | 0.256 | 0.236 | 0.225 | 0.230 | 0.170 |
| N100 vs. P300  N100 vs. RT-based | Lower | -0.234 | -0.096 | -0.162 | -0.072 | -0.225 | -0.370 | -0.045 | -0.248 |
|  | Upper | 0.109 | 0.268 | 0.267 | 0.292 | 0.137 | 0.042 | 0.326 | 0.143 |
| P300 vs. RT-based | Lower | -0.470 | 0.215 | -0.173 | -0.115 | -0.378 | 0.200 | 0.260 | 0.027 |
|  | Upper | 0.013 | 0.673 | 0.350 | 0.285 | 0.034 | 0.576 | 0.753 | 0.479 |

**Table A2.5**. Correlations between stage 1 scores for ANT indices and change scores (deltas) : 95% CIs.

|  |  | Stage 1 scores | | | |
| --- | --- | --- | --- | --- | --- |
| Delta |  | Baseline | Executive Control  Control | Alerting | Orienting |
| RT-based | Lower | -0.354 | -0.629 | -0.574 | -0.623 |
|  | Upper | 0.031 | -0.350 | -0.334 | -0.384 |
| N100 | Lower | -0.685 | -0.844 | -0.663 | -0.816 |
|  | Upper | -0.319 | -0.682 | -0.287 | -0.561 |
| P300 | Lower | -0.499 | -0.790 | -0.616 | -0.841 |
|  | Upper | 0.084 | -0.537 | -0.186 | -0.573 |

**Table A2.6**. Intercorrelations of two types of change score measure for RT-based indices: 95% CIs.

|  | | | Deltas | | | | Residuals | | | |
| --- | --- | --- | --- | --- | --- | --- | --- | --- | --- | --- |
|  | | | Baseline RT | Executive Control | Alerting | Orienting | Baseline RT | Executive Control | Alerting | Orienting |
| Deltas | Baseline RT | Lower |  |  |  |  |  |  |  |  |
|  |  | Upper |  |  |  |  |  |  |  |  |
|  | Executive Control | Lower | -0.209 |  |  |  |  |  |  |  |
|  |  | Upper | 0.160 |  |  |  |  |  |  |  |
|  | Alerting | Lower | 0.248 | -0.349 |  |  |  |  |  |  |
|  |  | Upper | 0.582 | 0.146 |  |  |  |  |  |  |
|  | Orienting | Lower | -0.068 | -0.027 | -0.174 |  |  |  |  |  |
|  |  | Upper | 0.273 | 0.466 | 0.242 |  |  |  |  |  |
| Res. | Baseline RT | Lower | 0.980 | -0.208 | 0.239 | -0.074 |  |  |  |  |
|  |  | Upper | 0.991 | 0.163 | 0.579 | 0.251 |  |  |  |  |
|  | Executive Control | Lower | -0.200 | 0.785 | -0.318 | -0.078 | -0.183 |  |  |  |
|  |  | Upper | 0.181 | 0.916 | 0.134 | 0.432 | 0.206 |  |  |  |
|  | Alerting | Lower | 0.310 | -0.267 | 0.832 | -0.080 | 0.338 | -0.235 |  |  |
|  |  | Upper | 0.598 | 0.196 | 0.932 | 0.229 | 0.629 | 0.188 |  |  |
|  | Orienting | Lower | -0.091 | -0.076 | -0.158 | 0.751 | -0.085 | -0.086 | -0.013 |  |
|  |  | Upper | 0.209 | 0.418 | 0.287 | 0.916 | 0.206 | 0.420 | 0.307 |  |

**Table A2.7**. Intercorrelations of two types of change score measure for N100: 95% CIs.

|  | | | Deltas | | | | Residuals | | | |
| --- | --- | --- | --- | --- | --- | --- | --- | --- | --- | --- |
|  | | | Baseline RT | Executive Control | Alerting | Orienting | Baseline RT | Executive Control | Alerting | Orienting |
| Deltas | Baseline | Lower |  |  |  |  |  |  |  |  |
|  |  | Upper |  |  |  |  |  |  |  |  |
|  | Executive Control | Lower | -0.287 |  |  |  |  |  |  |  |
|  |  | Upper | 0.103 |  |  |  |  |  |  |  |
|  | Alerting | Lower | -0.268 | -0.166 |  |  |  |  |  |  |
|  |  | Upper | 0.152 | 0.385 |  |  |  |  |  |  |
|  | Orienting | Lower | -0.129 | -0.137 | -0.254 |  |  |  |  |  |
|  |  | Upper | 0.246 | 0.387 | 0.255 |  |  |  |  |  |
| Res. | Baseline | Lower | 0.770 | -0.171 | -0.269 | -0.141 |  |  |  |  |
|  |  | Upper | 0.918 | 0.230 | 0.218 | 0.218 |  |  |  |  |
|  | Executive Control | Lower | -0.392 | 0.436 | -0.140 | -0.367 | -0.330 |  |  |  |
|  |  | Upper | 0.048 | 0.767 | 0.292 | 0.130 | 0.103 |  |  |  |
|  | Alerting | Lower | -0.235 | -0.155 | 0.770 | -0.310 | -0.249 | -0.085 |  |  |
|  |  | Upper | 0.229 | 0.357 | 0.932 | 0.228 | 0.290 | 0.308 |  |  |
|  | Orienting | Lower | -0.102 | -0.233 | -0.373 | 0.564 | -0.165 | -0.358 | -0.405 |  |
|  |  | Upper | 0.289 | 0.279 | 0.110 | 0.795 | 0.256 | 0.134 | 0.048 |  |

**Table A2.8**. Intercorrelations of two types of change score measure for P300: 95% CIs.

|  | | | Deltas | | | | Residuals | | | |
| --- | --- | --- | --- | --- | --- | --- | --- | --- | --- | --- |
|  | | | Baseline RT | Executive Control | Alerting | Orienting | Baseline RT | Executive Control | Alerting | Orienting |
| Deltas | Baseline | Lower |  |  |  |  |  |  |  |  |
|  |  | Upper |  |  |  |  |  |  |  |  |
|  | Executive Control | Lower | -0.043 |  |  |  |  |  |  |  |
|  |  | Upper | 0.341 |  |  |  |  |  |  |  |
|  | Alerting | Lower | -0.796 | -0.247 |  |  |  |  |  |  |
|  |  | Upper | -0.513 | 0.311 |  |  |  |  |  |  |
|  | Orienting | Lower | -0.164 | -0.091 | -0.447 |  |  |  |  |  |
|  |  | Upper | 0.519 | 0.368 | 0.504 |  |  |  |  |  |
| Res. | Baseline | Lower | 0.962 | -0.056 | -0.795 | -0.169 |  |  |  |  |
|  |  | Upper | 0.983 | 0.308 | -0.517 | 0.529 |  |  |  |  |
|  | Executive Control | Lower | -0.092 | 0.623 | -0.208 | -0.128 | -0.101 |  |  |  |
|  |  | Upper | 0.290 | 0.810 | 0.163 | 0.257 | 0.256 |  |  |  |
|  | Alerting | Lower | -0.816 | -0.276 | 0.841 | -0.496 | -0.839 | -0.188 |  |  |
|  |  | Upper | -0.552 | 0.226 | 0.949 | 0.482 | -0.576 | 0.132 |  |  |
|  | Orienting | Lower | -0.187 | -0.157 | -0.330 | 0.518 | -0.213 | -0.234 | -0.381 |  |
|  |  | Upper | 0.388 | 0.323 | 0.408 | 0.812 | 0.335 | 0.195 | 0.370 |  |

**Table 2.9**. Intercorrelations of N100 and P300 change scores, for two types of change score measure: 95% CIs.

|  | P300 | | | | | | | | |
| --- | --- | --- | --- | --- | --- | --- | --- | --- | --- |
|  |  | Deltas | | | | Residuals | | | |
| N100 |  | Baseline RT | Executive Control | Alerting | Orienting | Baseline RT | Executive Control | Alerting | Orienting |
| Baseline | Lower | -0.275 | -0.364 | -0.085 | -0.147 | -0.295 | -0.302 | -0.125 | -0.028 |
|  | Upper | 0.093 | 0.048 | 0.215 | 0.195 | 0.083 | 0.139 | 0.208 | 0.303 |
| Executive Control  Alerting | Lower | 0.063 | 0.283 | -0.333 | 0.065 | -0.140 | 0.242 | -0.327 | -0.148 |
|  | Upper | 0.504 | 0.625 | 0.218 | 0.520 | 0.454 | 0.593 | 0.246 | 0.255 |
| Alerting | Lower | -0.535 | -0.154 | 0.272 | -0.551 | -0.594 | -0.176 | 0.403 | -0.370 |
|  | Upper | 0.033 | 0.425 | 0.685 | 0.279 | -0.105 | 0.190 | 0.811 | 0.411 |
| Orienting | Lower | -0.243 | -0.048 | -0.330 | -0.035 | -0.260 | -0.288 | -0.410 | 0.037 |
|  | Upper | 0.312 | 0.316 | 0.197 | 0.475 | 0.274 | 0.232 | 0.082 | 0.474 |

**Table A2.10**. Correlations between RT-based indices and affect scales at stage 1 and for residualized change scores: 95% CIs.

|  |  | Stage 1 | | | | Residuals | | | |
| --- | --- | --- | --- | --- | --- | --- | --- | --- | --- |
|  |  | Baseline RT | Executive Control | Alerting | Orienting | Baseline RT | Executive Control | Alerting | Orienting |
| PA | Lower | -0.458 | -0.330 | -0.419 | -0.214 | -0.237 | -0.268 | -0.252 | -0.342 |
|  | Upper | -0.080 | 0.117 | -0.034 | 0.174 | 0.183 | 0.092 | 0.132 | 0.012 |
| NA | Lower | -0.102 | -0.006 | -0.173 | -0.040 | -0.215 | -0.241 | -0.318 | -0.317 |
|  | Upper | 0.344 | 0.484 | 0.239 | 0.317 | 0.165 | 0.138 | 0.302 | 0.126 |
