# Supplementary material for: Individual differences in brain attention networks: the challenge of indexing temporal change
Source: Front Cognit. 2025 Jun 18;4:1547773. doi: 10.3389/fcogn.2025.1547773 (PMC13281110; doi:10.3389/fcogn.2025.1547773)
Supplement: Supplementary file 3 [file Table_3.docx]

**Appendix 3: Associations Between Behavioral and ERP-Based Change Scores**

**Table A3.1.** Intercorrelations of RT-based and ERP-based residualized change score measures.

|  | N100 | | | | P300 | | | |
| --- | --- | --- | --- | --- | --- | --- | --- | --- |
| RT | Baseline | Executive Control | Alerting | Orienting | Baseline | Executive Control | Alerting | Orienting |
| Baseline | -.078 | .006 | -.057 | .119 | .012 | .106 | -.101 | .212^*^ |
| Executive Control | .211^*^ | -.098 | .078 | -.060 | -.059 | -.167 | .065 | .162 |
| Alerting | -.091 | .042 | .048 | .111 | -.121 | .073 | .060 | .031 |
| Orienting | .046 | -.061 | -.078 | -.085 | .028 | -.010 | -.070 | -.057 |

**p*<.05
